# Bystander defibrillation for out-of-hospital cardiac arrest in Ireland

**DOI:** 10.1016/j.resplu.2024.100712

**Published:** 2024-07-15

**Authors:** Tomás Barry, Alice Kasemiire, Martin Quinn, Conor Deasy, Gerard Bury, Siobhan Masterson, Ricardo Segurado, Andrew W. Murphy, Out-of-Hospital Cardiac Arrest Registry Steering Group

**Affiliations:** a School of Medicine, University College Dublin, Ireland; b Duke-NUS Medical School, Singapore; c UCD Centre for Support and Training in Analysis and Research, School of Public Health, Physiotherapy and Sports Science, University College Dublin, Dublin, Ireland; d Out-of-Hospital Cardiac Arrest Register, National Ambulance Service, Health Services Executive; e School of Medicine, University College Cork, Cork, Ireland; f University College Dublin, Ireland; g Clinical Strategy and Evaluation, Health Services Executive, National Ambulance Service, Ireland; h Discipline of General Practice, University of Galway, Galway, Ireland

**Keywords:** Resuscitation, Out-of-Hospital Cardiac Arrest, Cardiopulmonary Resuscitation, Bystander Defibrillation, Automatic External Defibrillators, Registry Data, Statistical Models, Public Health

## Abstract

**Aims:**

To describe and explore predictors of bystander defibrillation in Ireland during the period 2012 to 2020. To examine the relationship between bystander defibrillation and health system developments.

**Methods:**

National level Out of Hospital Cardiac Arrest (OHCA) registry data were interrogated, focusing on patients who had defibrillation performed. Bystander defibrillation (as compared to EMS initiated defibrillation) was the key outcome of concern. Logistic regression models were built and refined by fitting predictors, performing stepwise variable selection and by adding pairwise interactions that improved fit.

**Results:**

The data included 5,751 cases of OHCA where defibrillation was performed. Increasing year over time (OR 1.17, 95% CI 1.13, 1.21) was associated with increased adjusted odds of bystander defibrillation. Non-cardiac aetiology was associated with reduced adjusted odds of bystander defibrillation (OR 0.30, 95% CI 0.21, 0.42), as were increasing age in years (OR 0.99, 95% CI 0.987, 0.996) and night-time occurrence of OHCA (OR 0.67, 95% CI 0.53, 0.83). Six further variables in the final model (sex, call response interval, incident location (home or other), who witnessed collapse (bystander or not witnessed), urban or rural location, and the COVID period) were involved in significant interactions. Bystander defibrillation was in general less likely in urban settings and at home locations. Whilst women were less likely to receive bystander defibrillation overall, in witnessed OHCAs, occurring outside the home, in urban areas and outside of the COVID-19 period women were more likely, to receive bystander defibrillation.

**Conclusions:**

Defibrillation by bystanders has increased incrementally over time in Ireland. Interventions to address sex and age-based disparities, alongside interventions to increase bystander defibrillation at night, in urban settings and at home locations are required.

## Introduction

Ireland’s population of more than 5.1 million people^1^ experience approximately 3,000 OHCA (out-of-hospital cardiac arrest) resuscitation attempts each year, with 6–7% survival to hospital discharge.^2^ In 2021, 29% of all OHCA patients in Ireland had defibrillation performed and almost 7% of all OHCA patients had defibrillation attempted by bystanders.^2^ Previous research by our group has demonstrated that defibrillation by a bystander was the strongest predictor of overall OHCA survival in Ireland with an unadjusted odds ratio of 43.15 (95% CI, 34.85, 53.78).^3^ The prime importance of bystander defibrillation has been affirmed in the scientific literature which suggests that survival in patients with shockable OHCA rhythms decreases by 3–13% for each minute delay to defibrillation.^4^, ^5^ In Ireland, statutory EMS (emergency medical services) OHCA care is provided by the National Ambulance Service (NAS) and by Dublin Fire Brigade (DFB).^6^ All front-line EMS assets are equipped with a defibrillator and all EMS staff are trained to provide defibrillation. In addition to statutory EMS responders, Ireland also has an organised national voluntary network of community first responders (CFRs) who aim to provide CPR and when indicated defibrillation before EMS arrival.^6^ CFRs are dispatched by EMS control via mobile phone text messaging technology, generally travel to scene by car, and are routinely equipped with automatic external defibrillators (AEDs).^6^ Ireland does not yet have a central comprehensive register of AED locations.

Over the past decade the Irish health system has undergone several developments that are relevant to OHCA care. These developments are illustrated in supplementary Fig. 1 and have been previously discussed in detail.^7^, ^3^ Developments include policy initiatives, public education, first responder recruitment campaigns and EMS quality improvement initiatives. In terms of bystander defibrillation three key temporal events are worthy of specific note. In 2014, the Irish Health Information and Quality Authority published a health technology assessment of a proposed national public access defibrillation (PAD) programme for Ireland.^8^ This programme proposed legislation that identified a range of designated places where defibrillators would be installed and made available.^8^ Ultimately though, the health technology assessment found that the programme was unlikely to be cost effective as proposed. However, the authors concluded the program could potentially become cost effective with a 40% increase in AED utilisation when OHCAs occur in a public location and recommended the establishment of an EMS linked AED register.^8^ The proposed PAD legislation did not proceed although efforts to build a comprehensive EMS linked AED register are ongoing. In 2015 and 2016 there was a major national reconfiguration of EMS control that saw multiple regional centres consolidated to a single National Emergency Operations Centre (NEOC) and increased coordination and standardisation of OHCA response at national level.^7^ The year 2020 then heralded the arrival of the COVID-19 pandemic, with the initial wave occurring between February and July 2020.^7^ At this time widespread public health and social distancing measures were introduced to limit the spread of infection.^9^ During the COVID-19 pandemic international guidelines continued to recommend that OHCA bystanders attempt defibrillation.^10^ Despite this, COVID-19 was associated with disruption of OHCA care processes including bystander defibrillation and with worse survival outcomes across various international jurisdictions.^11^, ^12^ In Ireland, community first responder alerting was paused during the COVID pandemic owing to safety concerns. In other European countries some community responder schemes were deactivated however others continued to operate albeit often with decreased activity levels.^13^

The prime importance of defibrillation by bystanders in OHCA care is well established. Prior to this study though, no research had considered a temporal analysis of bystander defibrillation in Ireland. Thus, we aimed to interrogate national level OHCA registry data from the period 2012 to 2020, model predictors of bystander defibrillation, and explore the impact of relevant health system developments.

## Methods

### OHCAR – the Irish Out-of-Hospital Cardiac Arrest Register

The Irish Out-of-Hospital Cardiac Arrest Register (OHCAR) has collected national level data concerning OHCA resuscitation since 2012, employing the international ‘Utstein’ approach to data collection.^14^ Primary data sources are EMS dispatch and patient care records, with survival data being provided by receiving hospitals.^15^ OHCAR provides a key means of examining OHCA care including bystander defibrillation in Ireland over time.

### Study population

**Fig. 1:**
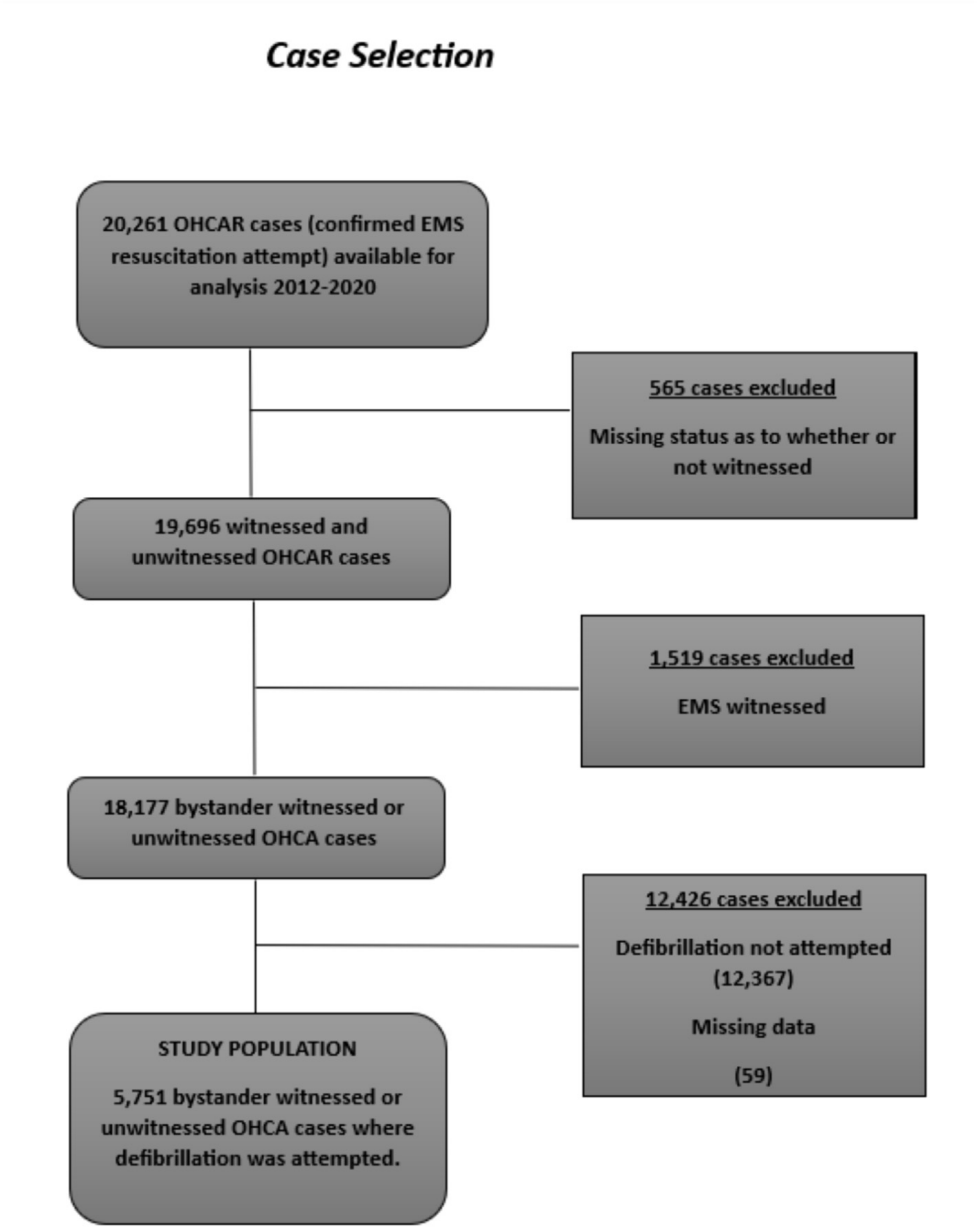
Bystander Defibrillation in Ireland 2012–2020: Case selection.

The population for this study were patients of all ages who had suffered un-witnessed, or bystander witnessed OHCA, and had defibrillation performed (Fig. 1). Patients who had defibrillation by bystanders were compared to those who had defibrillation initiated by EMS. In this study bystander defibrillation represented any defibrillation shock administered prior to EMS arrival. The time period of concern was January 1st 2012 to December 31st 2020.

### Research protocol development and variable selection

A detailed research protocol was developed in advance of data analysis.^7^ Several OHCAR variables were considered both potentially relevant to bystander defibrillation and noted as having adequate levels of data capture. The study team hypothesised that the centralisation of EMS control in 2015 and 2016 may have resulted in increased efficiencies in resource dispatch or telephone-based AED guidance and thus been associated with increased bystander defibrillation. In addition, given COVID-19′s disruptive effect on OHCA care process across different jurisdictions we hypothesised that the COVID-19 period might have been associated with a decrease in bystander defibrillation in Ireland.^7^ All analyses were based on the variables shown in supplementary Table 1. Variables 1–11 were obtained from OHCAR, whilst 12, 13 and 14 were created using the ‘year’ variable representing key component time periods that were considered potentially significant. Variable 10 was based on the Irish Central Statistics Office classification of urban or rural. When variables had multiple associated categories, these were collapsed to avoid decreased statistical power from analysis of an excessive number of potentially sparse categories. Year was treated as a continuous variable to conserve degrees of freedom and statistical power.

### Statistical model building

In conformity with our research protocol^7^ a series of logistic models were built to explore bystander defibrillation. Effect estimates for each individual variable in each model were summarised using odds ratios and 95% confidence intervals. Initially univariate logistic regression analysis was conducted. A full multiple logistic model was then fitted incorporating all predictor variables. Higher order (polynomial) terms were added to explore if the relationship between the outcome and continuous predictors was non-linear. Model refinement was conducted via stepwise model selection using the STEPAIC function in R. This involved building several models from all possible combinations of predictors by sequentially adding and dropping predictors and ultimately selecting the model with the lowest AIC (Akaike Information Criterion). This stepwise model was then further improved by adding pairwise interaction variables and retaining interactions which improved model fit. Each model (initial, stepwise, and with interactions) was then evaluated by AIC, model deviance and the result of the Hosmer-Lemeshow Goodness of Fit (GOF) test. The predictive ability of the final, best fitting model was evaluated using 10-fold cross-validation.

### Missing data & sensitivity analysis

The proportions of missing data for all variables were documented and evaluated. The mice package in R was used to conduct missing data sensitivity analyses via multiple imputation. This involved multivariate imputation by chained equations using ten imputed data sets and methods appropriate for each variable (binomial or polynomial logistic, or linear regressions). Following this, results from complete case analysis and multiple imputation were inspected and compared.

### Ethical approval

The National University of Ireland Galway, Research Ethics Committee provided ethical approval for this study in advance of data processing and analysis (Reference 2020.01.012; Amend 2106).

## Results

**Fig. 2:**
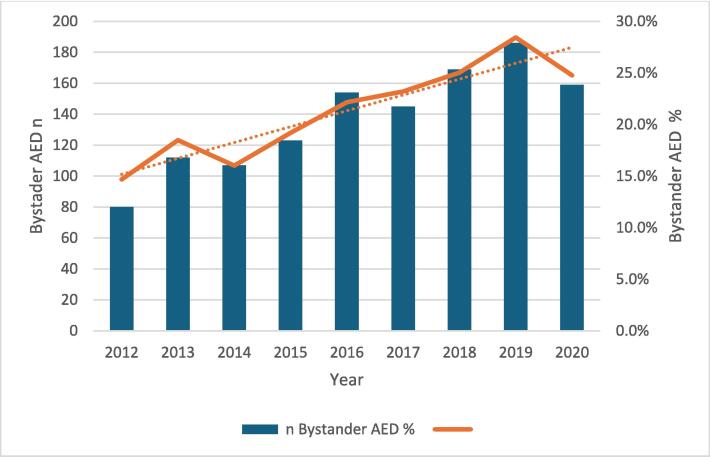
Bystander Defibrillation in Ireland 2012–2020, absolute number and proportion of all patients who had defibrillation performed over time.

The data contained a total of 5,751 cases. Missing data across predictor variables ranged from 0.0% to 4.2% (see Table 1a, Table 1b). Fig. 2 illustrates the absolute number and relative proportion of cases with bystander defibrillation (as compared to EMS initiated defibrillation) over the study period. It demonstrates the overall number and proportion of cases with bystander defibrillation increased over time. Table 1a summarises the proportion of bystander defibrillation across various predictor variable categories. Table 1b provides a summary of continuous predictor variables for all cases, cases where bystander defibrillation was performed and in cases where EMS initiated defibrillation. Of all patients (i.e. those OHCA patients who had defibrillation performed) the overall proportion of bystander defibrillation was 21.5%. The highest unadjusted rate of bystander defibrillation (36.5%) was at locations other than home. The lowest rate was in OHCA presumed not to be of cardiac origin (11.7%). Median age (65.0 versus 66.0 years) was lower whereas median call response interval (15.0 versus 11.0 min) was higher in patients who had bystander defibrillation. Supplementary Table 2 presents the results of univariate, unadjusted analysis.

**Table 1a:** Bystander Defibrillation in Ireland in Ireland 2012–2020: Summary of categorical variables.

Variable	Available	Missing		Categories			Outcome Available	Bystander AED	
	*n*	*n*	%		*n*	%	*n*	*n*	%
Who delivered first Shock	5751	0	0.0%	Bystander AED	1235	21.5%			
				EMS Defibrillation	4516	78.5%			
				Total	5751				
Aetiology	5751	0	0.0%	Presumed Cardiac	5299	92.1%	5299	1182	22.3%
				Presumed Other	452	7.9%	452	53	11.7%
				Total	5751	100.0%			
Gender	5746	5	0.1%	Male	4389	76.4%	4389	1013	23.1%
				Female	1357	23.6%	1357	221	16.3%
				Total	5746	100.0%			
Incident location	5726	25	0.4%	Other Location	2244	39.2%	2244	820	36.5%
				Home Location	3482	60.8%	3482	412	11.8%
				Total	5726	100.0%			
Season	5751	0	0.0%	Winter	2963	51.5%	2963	616	20.8%
				Other	2788	48.5%	2788	619	22.2%
				Total	5751	100.0%			
Time of Day	5751	0	0.0%	Morning	2463	42.8%	2463	575	23.3%
				Evening	2204	38.3%	2204	522	23.7%
				Night	1084	18.8%	1084	138	12.7%
				Total	5751	100.0%			
Who Witnessed Collapse	5751	0	0.0%	Bystander Witnessed	4306	74.9%	4306	1025	23.8%
				Not Witnessed	1445	25.1%	1445	210	14.5%
				Total	5751	100.0%			
Urban or Rural	5512	239	4.2%	Urban Location	3694	67.0%	3694	684	18.5%
				Rural Location	1818	33.0%	1818	485	26.7%
				Total	5512	100.0%			
Weekday or Weekend	5751	0	0.0%	Weekday	3976	69.1%	3976	888	22.3%
				Weekend	1775	30.9%	1775	347	19.5%
				Total	5751	100.0%			
Transition Period (2015 & 2016)	5751	0	0.0%	Other	4415	76.8%	4415	958	21.7%
				Transition Period	1336	23.2%	1336	277	20.7%
				Total	5751	100.0%			
Post Transition (2017–2020)	5751	0	0.0%	Other	3155	54.9%	3155	576	18.3%
				Post Transition Period	2596	45.1%	2596	659	25.4%
				Total	5751	100.0%			
Covid Period (2020)	5751	0	0.0%	Other	5109	88.8%	5109	1076	21.1%
				Covid Period	642	11.2%	642	159	24.8%
				Total	5751	100.0%

**Table 1b:** *Bystander Defibrillation in Ireland* in Ireland 2012–2020: Summary of continuous variables.

Variable	Available Cases	Missing		All		Bystander AED		EMS Defibrillation	
		*n*	%	Mean	SD	Mean	SD	Mean	SD
				*Median*	*IQR*	*Median*	*IQR*	*Median*	*IQR*
Age (Years)	5659	92	1.6	**63.4**	**17.0**	**63.1**	**16.4**	**63.5**	**17.2**
				66.0	54.0–76.0	65.0	54.0–75.0	66.0	53.0–76.0
Call Response (Mins)	5610	141	2.5	**14.0**	**8.0**	**17.0**	**9.0**	**13.0**	**8.0**
				12.0	8.0–19.0	15.0	9.0–22.0	11.0	8.0–18.0

Supplementary Table 3 and Table 2 present the results of the multivariable, adjusted analysis. During modelling quadratic terms for continuous variables were non-significant. This indicated that there was no substantial nonlinear relationship between the continuous predictors and the binary outcome on the log scale. Supplementary Table 3 summarises the results of the full and stepwise modelling. In moving from the full to stepwise model the variables transition period, post transition period and season were dropped from the model. The model obtained from the stepwise procedure was then further refined by adding interactions that improved model fit. The results of this final model are summarised in Table 2. Model fit statistics (deviance and AIC) improved from full to final model. In addition, the Hosmer-Lemeshow test revealed no model with inadequate fit. The pseudo R2 of the final model was 24.1%. Tenfold cross validation was used to assess the predictive ability of the final model which was found to be 79.5%. The outputs of multiple imputation sensitivity analysis are included in Table 2. In general, complete case and multiple imputation analysis yielded similar results. Of note ‘weekend’ (OR 0.82 95%CI 0.71, 0.96) was associated with decreased odds of bystander defibrillation in multiple imputation analysis.

**Table 2:** *Bystander Defibrillation in Ireland* in Ireland 2012–2020: Multivariable analysis, Final Model with Interactions – Complete Cases and Multiple Imputation.

Predictor	Involved in Interactions	Complete Case		Multiple Imputation	
		Odds Ratio (95% Confidence Interval)	*p*-value	Odds Ratio (95% Confidence Interval)	*p*-value
Other Aetiology		0.297 (0.205, 0.421)	<0.001	0.319 (0.230, 0.444)	<0.001
Age (years)		0.992 (0.987, 0.996)	<0.001	0.993 (0.988, 0.997)	0.001
Female	*	1.968 (1.346, 2.874)	<0.001	1.866 (1.297, 2.685)	0.001
Call Response Interval (minutes)	*	1.051 (1.037, 1.066)	<0.001	1.053 (1.038, 1.067)	<0.001
Home Location	*	0.108 (0.077, 0.150)	<0.001	0.122 (0.089, 0.169)	<0.001
Year		1.166 (1.125, 1.208)	<0.001	1.162 (1.123, 1.202)	<0.001
Evening		1.059 (0.905, 1.238)	0.476	1.063 (0.916, 1.235)	0.42
Night		0.665 (0.528, 0.833)	<0.001	0.642 (0.515, 0.800)	<0.001
Not Witnessed	*	0.699 (0.521,0.931)	0.016	0.657 (0.499, 0.867)	0.003
Rural Location	*	1.770 (1.260, 2.486)	0.001	1.803 (1.293, 2.514)	<0.001
Weekend		0.865 (0.737, 1.015)	0.077	0.822 (0.705, 0.959)	0.013
Covid Period (2020)	*	0.986 (0.746, 1.302)	0.922	0.937 (0.716, 1.226)	0.634
Call Response Interval * Female		0.968 (0.949, 0.986)	0.001	0.969 (0.951, 0.987)	<0.001
Call Response Interval * Home Location		1.024 (1.008, 1.041)	0.004	1.020 (1.004, 1.037)	0.015
Call Response Interval * Rural Location		0.982 (0.966, 0.998)	0.03	0.985 (0.969, 1.001)	0.067
Female * Home Location		0.682 (0.468, 0.991)	0.046	0.702 (0.490, 1.006)	0.054
Rural Location * Not Witnessed		0.586 (0.402, 0.852)	0.005	0.576 (0.398, 0.832)	0.003
Home Location * Rural Location		1.410 (1.020, 1.948)	0.037	1.294 (0.943, 1.777)	0.11
Female * Covid Period		0.259 (0.122, 0.510)	<0.001	0.309 (0.159, 0.599)	0.001
Home Location * Not Witnessed		1.703 (1.180, 2.457)	0.004	1.801 (1.267, 2.559)	0.001

Fig. 3a, Fig. 3b together provide an overall summary of the final model. Fig. 3a illustrates the (adjusted) odds ratios and associated 95% confidence intervals for the final model predictors that were not involved in interactions. In this adjusted model non-cardiac aetiology was associated with reduced adjusted odds of bystander defibrillation (OR 0.30, 95% CI 0.21, 0.42), as were age (OR 0.99, 95% CI 0.987, 0.996) and night-time (OR 0.67, 95% CI 0.53, 0.83). Increasing year (OR 1.17, 95% CI 1.13, 1.21) was associated with increased adjusted odds of bystander defibrillation. Fig. 3b summarises the final model predictor effects for those predictors that were involved in interactions. Fig. 3b compares effect estimates considering a base case scenario with other relevant interaction states. The baseline comparator status for this analysis represents the theoretical scenario of presumed cardiac aetiology, male sex, other than home location, daytime, witnessed, urban location, weekday and not the COVID period. Fig. 3b illustrates that for this relevant population the odds of bystander defibrillation were increased when the event occurred in a rural location (OR 1.77 95%CI 1.26, 2.49). An interaction with un-witnessed status however then modified the effect estimate downwards (OR 1.04 95% CI 0.66, 1.64), rendering the association with rural location insignificant. Home location was associated with reduced odds of bystander defibrillation (OR 0.11 95% CI 0.08, 0.15) which was further modified by an interaction with rural location (OR 0.15 95%CI 0.10, 0.23) and unwitnessed status (OR 0.18, 95% CI 0.12, 0.28). Female sex was associated with increased odds of bystander defibrillation (OR 1.97 (95% CI 1.35, 2.87) which was modified by interactions with home location (OR 1.34 95% CI 0.87, 2.06) and the COVID period (OR 0.35 95% CI 0.15, 0.73). Call response interval in minutes was associated with increased odds of bystander defibrillation (OR 1.05 95%CI 1.04, 1.07) which was modified by female sex (OR 1.02 95%CI 0.997, 1.04), rural (OR 1.03, 95% CI 1.02, 1.05) and home location (OR 1.08 95%CI 1.06, 1.09).

**Fig. 3a:**
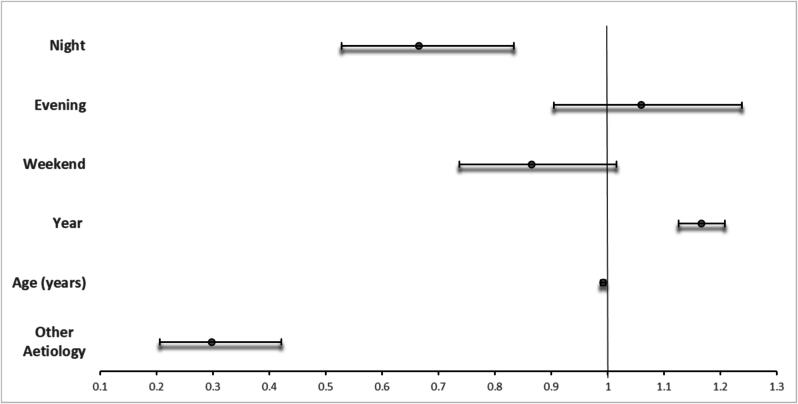
Bystander Defibrillation *in Ireland* in Ireland 2012–2020: Multivariable analysis, Final model − Predictors without Interactions (odds ratios & 95% confidence intervals – interval scale).

**Fig. 3b:**
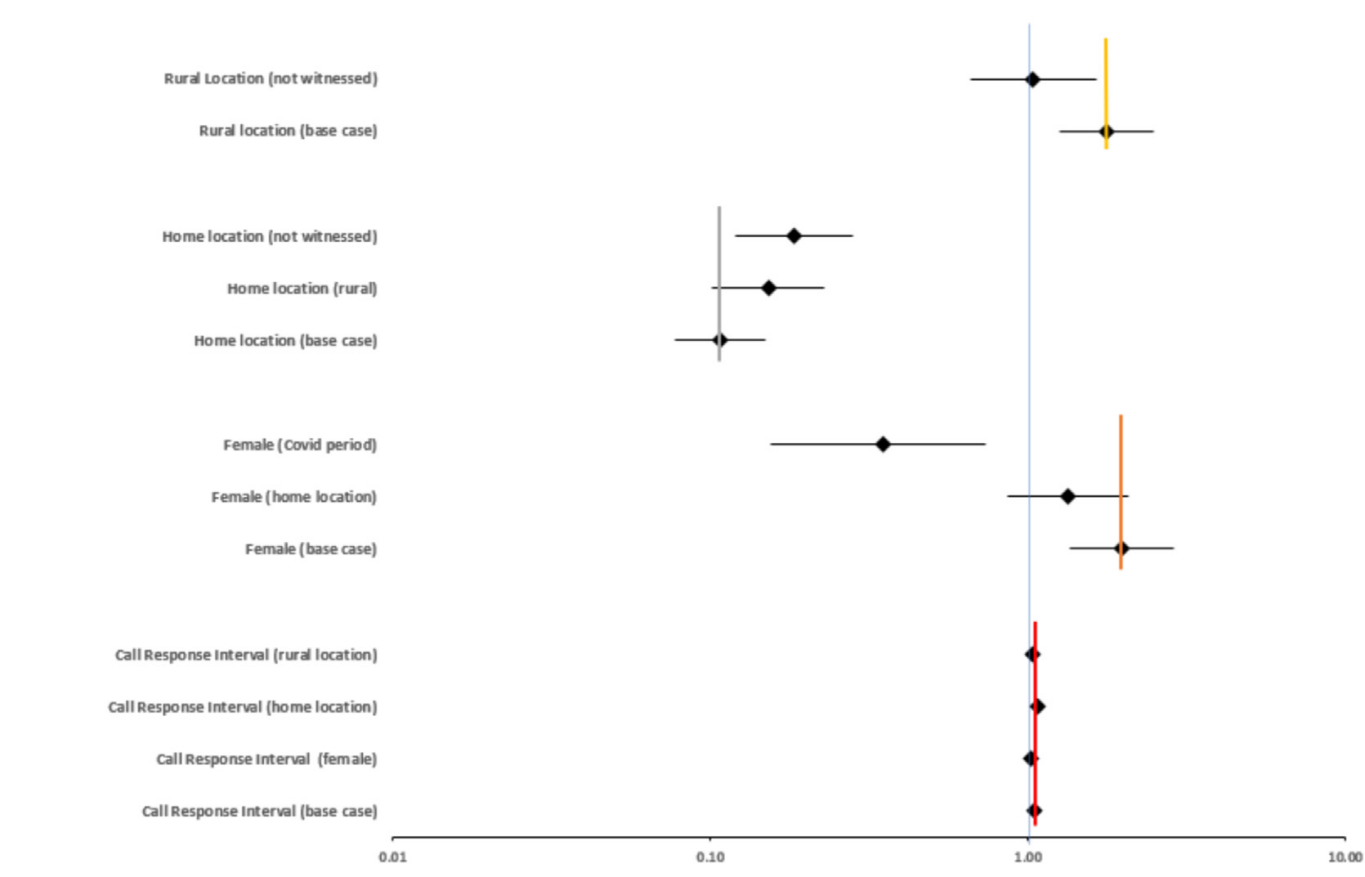
*Bystander Defibrillation in Ireland* in Ireland 2012–2020: Multivariable analysis, Final model predictors with interactions (odds ratio & 95% confidence intervals – log scale). Base case refers to the relevant variable with other model variables at baseline comparator status i.e. presumed cardiac aetiology, male sex, other than home location, daytime, witnessed, urban location, weekday and not the COVID period.

## Discussion

This research incorporated nine years of national out-of-hospital cardiac arrest registry data and included 5,751 patients who had defibrillation performed. In general, bystander defibrillation increased incrementally over the period. The adjusted odds of bystander defibrillation demonstrated an increase of 17% on average per year (OR 1.17 95%CI 1.13, 1.21). This supports the assertion that system level developments, including the expansion of AED equipped first responders are likely to have contributed to increased bystander defibrillation over the period of concern. It is also possible that general awareness and availability of AEDs increased in the wider community over the period. Unfortunately, the data collected by OHCAR does not allow bystander defibrillation by EMS dispatched first responders to be delineated from that provided by opportunistic bystanders. Interestingly, the transition to a single national control centre in 2015/6 was not associated with a significant increase in bystander defibrillation when other important variables were adjusted for.

In the final model a non-cardiac aetiology (OR 0.30, 95% CI 0.21, 0.42), age in years (OR 0.99, 95% CI 0.987, 0.996) and night-time (OR 0.67, 95% CI 0.53, 0.83) were associated with reduced adjusted odds of bystander defibrillation. OHCA that occurred at the weekend was associated with reduced adjusted odds of bystander defibrillation in sensitivity analysis (OR 0.82, 95%CI 0.71, 0.96). OHCA of presumed non-cardiac aetiology represented a minority of cases (less than 8%) in this study. Nonetheless it is important to ensure that in such circumstances an AED is attached, as if defibrillation is indicated it can make a critical contribution to care.^16^ The observation that the odds of bystander defibrillation decreased with age warrants consideration. Parallel research by our group has also found that both the adjusted odds of bystander CPR and OHCA survival decreased with age.^3^, ^17^ Thus, whether older OHCA patients face delays or barriers to bystander care, and potentially suffer decreased survival consequently should be explored. It is perhaps not surprising that the adjusted odds of bystander defibrillation were lower at nighttime (23.00–06.59) as both OHCA patients and AEDs may be less accessible during this time window. Given that in this study 20% of OHCA involving defibrillation occurred at nighttime, it is important to consider whether wider availability of home-based AEDs coupled with technologies that enhance early detection of OHCA could contribute to addressing this disparity in future. For instance, it has been suggested that ‘smart speaker’ technology may be able to identify audible biomarkers of OHCA and summon assistance, while smart apps and drone technology or indeed low cost home AEDs could facilitate bystander defibrillation before EMS arrival.^18^ Over 30% of OHCA in this study occurred at the weekend where sensitivity analysis of the final model, and the stepwise model demonstrated decreased odds of bystander defibrillation. This could possibly relate to accessibility of AEDs at the weekend and again warrants further study.

In the final model several key variables were involved in significant interactions (sex, call response interval, incident location (home or other), who witnessed collapse (bystander or not witnessed), urban or rural location, and the COVID period) highlighting key differences in the likelihood of bystander defibrillation across important subgroups of OHCA patients. Fig. 3b summarises these differences by considering a baseline population with witnessed OHCA, occurring during the daytime, of presumed cardiac aetiology, who were male sex, at locations other than home, in urban areas, excluding the COVID period. For this population a shift to rural location significantly increased the odds of bystander defibrillation (OR 1.77, 95% CI 1.26, 2.49). Indeed, in the full and stepwise models that also adjusted for covariates but without including interaction terms, rural location was also associated with increased odds of bystander defibrillation. Parallel work by our group has also noted increased odds of bystander CPR in rural areas.^17^ Given the known importance and synergy of bystander CPR and defibrillation, there may be potential to increase OHCA survival in Ireland by focusing on increasing these important aspects of the chain of survival in urban areas. When OHCA occurred at home this was associated with reduced odds of bystander defibrillation (OR 0.11, 95%CI 0.08, 0.15) and although this effect estimate was modified by interactions with unwitnessed status and rural location, remained associated with decreased odds of bystander defibrillation. Furthermore, in the full and stepwise models home location was associated with markedly decreased odds of bystander defibrillation. Over 60% of all OHCA in this current study occurred at home. Thus, interventions that can increase bystander defibrillation at home locations are likely to hold significant potential to increase overall OHCA survival in Ireland. Previous research from Denmark found low AED coverage for OHCA at residential locations and the authors suggested that implementing first-responder programs could increase the proportion of OHCA patients at home that could receive bystander defibrillation.^19^

In univariate analysis (OR 0.65 95% CI 0.55, 0.76), the full logistic model (OR 0.83 95%CI 0.69, 0.99) and the stepwise model without interactions (OR 0.83 95%CI 0.69, 0.99) female sex was associated with decreased odds of bystander defibrillation. However, in the relevant comparator group in the final model with interactions, female sex was associated with increased odds of bystander defibrillation (OR 1.97 95% CI 1.35, 2.87). This effect estimate was modified to an odds ratio of 1.34 (95%CI 0.87, 2.06) when OHCA occurred at home and 0.35 (95%CI 0.15, 0.73) during the COVID period. This suggests that whilst women were less likely to receive bystander defibrillation overall, in witnessed OHCAs, occurring outside the home, in urban areas and outside of the COVID-19 period women were more likely to receive bystander defibrillation. Previous research has demonstrated that women are less likely to receive bystander defibrillation and suggested that public awareness initiatives are necessary to support AED usage in this population.^20^ Such initiatives would appear to have some relevance also in the Irish context.

### Limitations

This research is observational in nature and cannot definitively establish whether the predictors studied directly influenced bystander defibrillation. Following the approach set out in our protocol^7^ our modelling strategy undertook a statistically driven exploratory approach. It is thus important to acknowledge the limitations of stepwise variable selection and consider the possibility that effect sizes may be inflated, and p-values too liberal. We thus have not made claims of effect based on statistical significance and interpret effect sizes and confidence intervals with some caution. The OHCAR data did not capture who performed bystander defibrillation and thus we could not delineate between bystander defibrillation performed by organised community first responders and that performed by opportunistic bystanders. In this study bystander defibrillation was compared to defibrillation initiated by EMS which included patients who initially presented with non-shockable rhythms but transitioned to a shockable rhythm during resuscitation. Further research that focuses on the Utstein (bystander witnessed and initial shockable rhythm) comparator in Ireland is ongoing and will yield further insights into bystander defibrillation in this important cohort.

## Conclusions

Bystander defibrillation increased incrementally during 2012 to 2020 in Ireland. Overall, women were less likely to receive bystander defibrillation, although were more likely in witnessed OHCA, occurring outside the home, in urban areas. Bystander defibrillation was less likely in urban locations, even after adjusting for important covariates. Bystander defibrillation was also less likely at home locations. Significant proportions of OHCA where defibrillation is performed occur at home and in urban locations. Thus, increasing bystander defibrillation at these locations should be targets for intervention in Ireland.

## CRediT authorship contribution statement

**Tomás Barry:** Writing – review & editing, Writing – original draft, Resources, Methodology, Investigation, Funding acquisition, Formal analysis, Conceptualization. **Alice Kasemiire:** Formal analysis. **Martin Quinn:** Data curation, Writing – review & editing. **Conor Deasy:** Writing – review & editing, Writing – original draft, Methodology. **Gerard Bury:** Writing – review & editing, Writing – original draft, Conceptualization. **Siobhan Masterson:** Writing – review & editing, Writing – original draft, Methodology, Investigation, Funding acquisition, Formal analysis, Data curation, Conceptualization. **Ricardo Segurado:** Writing – review & editing, Writing – original draft, Validation, Methodology, Investigation, Formal analysis. **Andrew W. Murphy:** Writing – review & editing, Writing – original draft, Resources, Methodology, Formal analysis, Data curation.

## Declaration of competing interest

The authors declare the following financial interests/personal relationships which may be considered as potential competing interests: ‘TB has research, clinical and educational roles in resuscitation care. He is a member of the Pre-Hospital Emergency Care Council (Ireland). All authors declare no conflict of interest’.
