# Supplementary material for: Bystander defibrillation for out-of-hospital cardiac arrest in Ireland
Source: Resusc Plus. 2024 Jul 15;19:100712. doi: 10.1016/j.resplu.2024.100712 (PMC11304059; doi:10.1016/j.resplu.2024.100712)
Supplement: Supplementary Data 1 [file mmc1.docx]

**Supplementary Figure 1:** Resuscitation for out-of- hospital cardiac arrest in Ireland 2012 -2020: Summary of health system temporal developments

From - Barry T, Kasemiire A, Quinn M et al. Outcomes of out-of-hospital cardiac arrest in Ireland 2012-2020: Protocol for an observational study [version 2; peer review: 3 approved, 1 approved with reservations]. HRB Open Res 2023, 6:17 (<https://doi.org/10.12688/hrbopenres.13699.2>)

CFR: Community first responder, CPR: Cardio pulmonary resuscitation, GP: General Practitioner, NAS: National Ambulance Service, OHCA: Out-of-hospital cardiac arrest, PAD: Public access defibrillation,


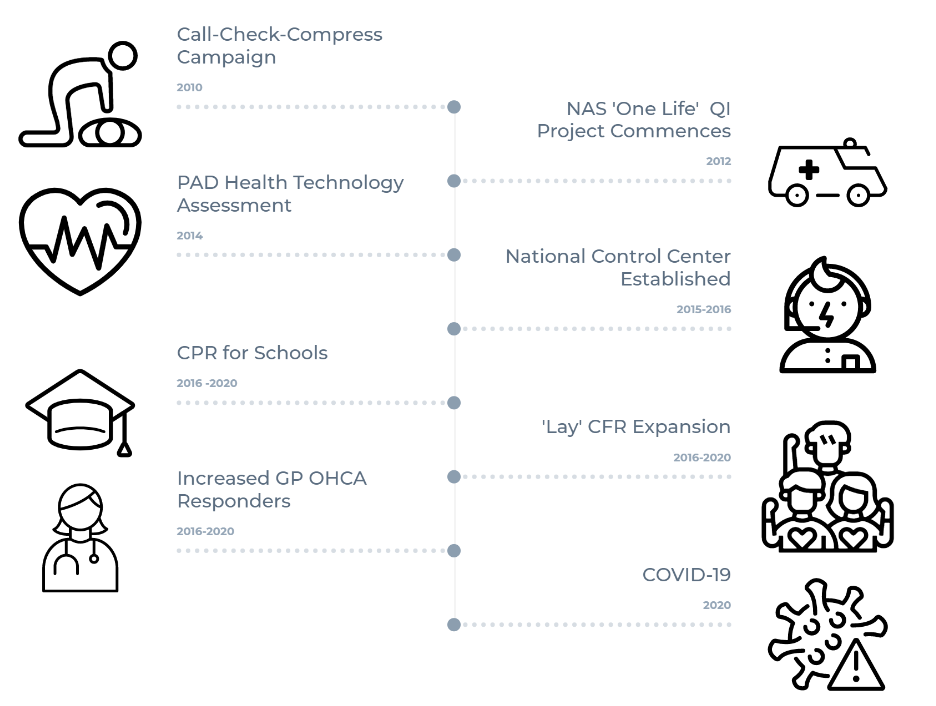


***Supplementary Table 1:*** *Bystander Defibrillation in Ireland 2012 -2020: Summary of variables for analysis*

***Supplementary T******able 2:*** *Bystander Defibrillation in Ireland in Ireland 2012 -2020: Univariate analysis*

| **Predictor** | **Univariate Model** | |
| --- | --- | --- |
|  |  |  |
|  | Odds Ratio (95% Confidence Interval) | p-value |
|  |  |  |
| Other Aetiology | 0.463 (0.341, 0.615) | <0.001 |
| Age (years) | 0.998 (0.995, 1.002) | 0.409 |
| Female | 0.648 (0.551, 0.759) | <0.001 |
| Call Response Interval (minutes) | 1.038 (1.031, 1.045) | <0.001 |
| Home Location | 0.233 (0.204, 0.266) | <0.001 |
| Not Winter (April-Sept) | 1.087 (0.959, 1.233) | 0.192 |
| Year | 1.097 (1.069, 1.125) | < 0.001 |
| Evening | 1.019 (0.889, 1.167) | 0.785 |
| Night | 0.479 (0.390, 0.584) | <0.001 |
| Not Witnessed | 0.544 (0.462, 0.639) | <0.001 |
| Rural Location | 1.601 (1.401, 1.829) | <0.001 |
| Weekend | 0.845 (0.735, 0.970) | 0.018 |
| *Transition Period (2015 & 2016) | 1.330 (1.109, 1.594) | 0.002 |
| *Post Transition Period (2017-2020) | 1.730 (1.485, 2.014) | < 0.001 |
| COVID Period (2020) | 1.234 (1.019, 1.494) | 0.031 |
|  |  |  |

*Dummy variables adjusted for each other

***Supplementary Table 3:*** *Bystander Defibrillation in Ireland in Ireland 2012 -2020: Multivariable analysis, Full and Stepwise Models*
